# Insights into structure and activity of natural compound inhibitors of pneumolysin

**DOI:** 10.1038/srep42015

**Published:** 2017-02-06

**Authors:** Hongen Li, Xiaoran Zhao, Xuming Deng, Jianfeng Wang, Meng Song, Xiaodi Niu, Liping Peng

**Affiliations:** 1 Department of Respiratory Medicine, The First Hospital of Jilin University, Key Laboratory of Zoonosis, Ministry of Education, Department of Food Quality and Safety, College of Veterinary Medicine, Jilin University, Changchun, China

## Abstract

Pneumolysin is the one of the major virulence factor of the bacterium *Streptococcus pneumoniae*. In previous report, it is shown that β-sitosterol, a natural compound without antimicrobial activity, is a potent antagonist of pneumolysin. Here, two new pneumolysin natural compound inhibitors, with differential activity, were discovered via haemolysis assay. To explore the key factor of the conformation for the inhibition activity, the interactions between five natural compound inhibitors with differential activity and pneumolysin were reported using molecular modelling, the potential of mean force profiles. Interestingly, it is found that incorporation of the single bond (C22-C23-C24-C25) to replace the double bond (hydrocarbon sidechain) improved the anti-haemolytic activity. In view of the molecular modelling, binding of the five inhibitors to the conserved loop region (Val372, Leu460, and Tyr461) of the cholesterol binding sites led to stable complex systems, which was consistent with the result of β-sitosterol. Owing to the single bond (C22-C23-C24-C25), campesterol and brassicasterol could form strong interactions with Val372 and show higher anti-haemolytic activity, which indicated that the single bond (C22-C23-C24-C25) in inhibitors was required for the anti-haemolytic activity. Overall, the current molecular modelling work provides a starting point for the development of rational design and higher activity pneumolysin inhibitors.

*Streptococcus pneumoniae* is an important bacterial pathogen that causes many infections, for example, otitis media pneumonia, meningitis and bacteraemia. Such infections have led to approximately 2 million deaths, including 1 million children under 5 years old. Pneumococcal virulence factors play a critical role in its pathogenicity, especially pneumolysin (PLY). PLY, which belongs to the cholesterol-dependent cytolysin (CDC) protein family^1^, is a 53 kD haemolytic toxin whose activity requires the presence of cholesterol on the cell membranes. Since PLY has been well characterized as the key cause of alveolar oedema and haemorrhage during pneumococcal pneumonia and is known to interact with cells of the respiratory system^2^, PLY has been regarded as an important candidate drug target for the treatment of pneumonia infection.

Recently, studies on the pathogenic mechanism of PLY have been reported frequently. Prior to 1998, the 3D structure of perfringolysin O (PFO) has been used as an accurate model to study the monomeric structure of PLY due to their high sequence homology^3^. It has been shown that a conserved tryptophan-rich motif in domain 4 is thought to interact with cholesterol and is essential for cytolytic activity. Furthermore, studies with PFO show that monomers can bind to the membrane (based on PFO’s interaction with cholesterol) and oligomerize on the surface to form a prepore ring, which leads to the cell rupture^4^,^5^,^6^,^7^. During pore formation, a study on the conformation transition from monomeric to oligomeric structure reveals that domain 3 is expulsed from its original position in the monomer during oligomerization, and the base of domain 4 contacts the bilayer along with the extension of domain 3^8^. Similar conclusions also emerged regarding PLY. Domain 4 is essential for the initial binding to membranous cholesterol, and this interaction leads to subsequent membrane damage^9^. The study on the interaction of PLY with cholesterol has been systematically reported^10^. Through a series of biophysical studies, cholesterol can strongly bind to the conserved undecapeptide loop region in domain 4. In 2005, a clear view of the PLY pore structure was determined with the atomic structure, and the pore-forming mechanism has been shown using the cryo-EM maps method^11^. One study shows that many aspects can impact the progress of pore formation, including the buckling of domain 2, the massive refolding of domain 3, and the interface between domains 2 and 4. However, there are few reports on the study of antimicrobial activities using PLY as a drug target. Due to its strong binding with PLY, cholesterol has been used as a first-line inhibitor for the treatment of pneumococcal keratitis^12^. Only in 2014 have studies reported the development of a PLY antibody and vaccine^13^,^14^.

In our previous work, it is reported for the first time that β-sitosterol (BSS), a natural plant-derived steroid alcohol, can bind to PLY directly by interacting with the cholesterol binding site^15^. Due to the competitive binding, BSS can inhibit the lytic activity of PLY and prevent cell injury. Unfortunately, the information about the relationship between this activity and the molecular structure of PLY was unclear. Here, the five pneumolysin natural compound inhibitors with differential activity were discovered via haemolysis assay. Based on the methods of molecular dynamics (MD) simulations and free energy calculations, the binding of the five inhibitors to the conserved loop region of the cholesterol binding sites led to stable complex systems, which was consistent with the results of β-sitosterol binding. Via molecular modelling, the mechanism of the haemolytic activity of inhibitors and the structure-activity relationships of these inhibitors was investigated.

## Results

### Inhibition of the haemolytic activity of pneumolysin by different sterols

**Figure 1:**
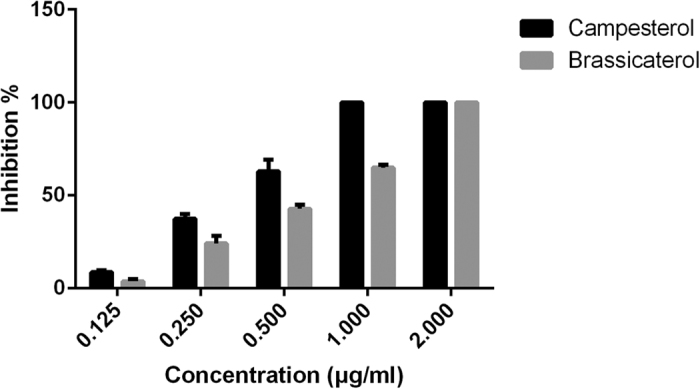
Inhibition of the haemolytic activity of pneumolysin by campesterol and brassicasterol. Blocking the cytotoxic effect of 1 μg pneumolysin needs either 1 μg campesterol or 2 μg brassicasterol.

Although the structure of these five nature sterols were highly analogous, the anti-haemolytic activity of pneumolysin was totally different. We have reported that to completely neutralize 1 μg of purified pneumolysin, 1 μg cholesterol (CHO), 1 μg β-sitosterol (SIO) or 32 μg stigmasterol (SIG) is required^15^. In this study, we found that either 1 μg campesterol (CAM) or 2 μg brassicasterol (BRA) was needed to block the cytotoxic effect of 1 μg of pneumolysin cytotoxic effect (Fig. 1). Fruthermore, the MICs of these five natural steroids for *S. pneumoniae* D39 were all greater than 1024 μg/ml, suggesting that no antibacterial acitivity was observed for these compounds.

### Confirmation of the binding mode of inhibitors with PLY

**Figure 2:**
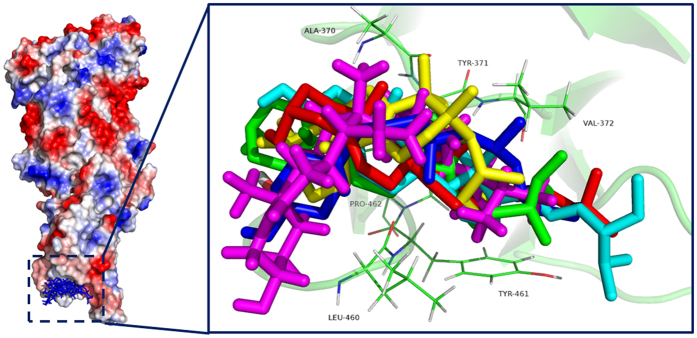
The RMSD values of the protein during the MD simulations of PLY with CHO, CAM, SIO, BRA and SIG. Different colours show the different RMSD values of PLY with CHO, CAM, SIO, BRA and SIG.

Based on the functional analyses, it is suggested that the five natural compounds can inhibit the lytic activity of PLY via direct binding with PLY. To determine the conformational properties of PLY in complex with the inhibitors, theoretical calculations were performed for the complex systems. Using 100-ns standard molecular modelling, a stable structure of the complexes was obtained. As shown in Fig. 2, the RMSD values of each PLY-inhibitor complex equilibrated at 20 ns of simulation, which indicated that each structure could reach the relative stability after the initial 20 ns, and the final 80 ns of the MD simulation could be used for the next analysis. Furthermore, Fig. 2 clearly shows that the RMSD values of the PLY-CHO, PLY-CAM, PLY-SIO and PL-BRA complexes have weaker changes (~0.1 nm) compared to the ~0.35 nm change of the PLY-SIG complex, which indicated that the binding of PLY with CHO, CAM, SIO and BRA were stronger than SIG.

**Figure 3:**
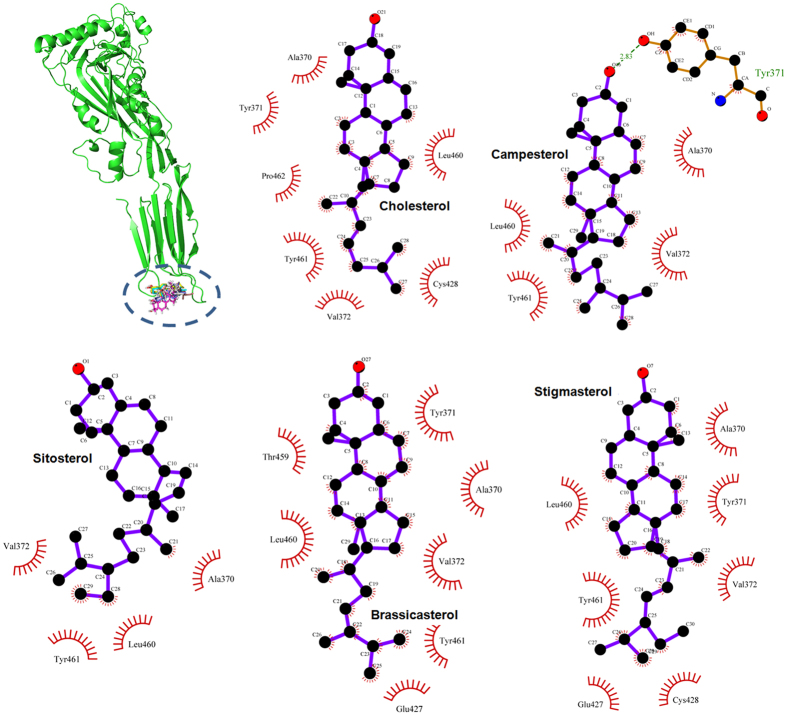
The binding modes of the five inhibitors with PLY are shown via the LigPlot program. The binding modes of the five inhibitors with PLY were the same, and the stabilization of the PLY-inhibitor complexes was mostly due to residues Tyr371, Val372, Leu460, and Tyr461.

As shown in Fig. 3, the MD simulations generated reliable models for the PLY-inhibitor complexes. It has been shown that inhibitors could bind to the loop region of domain 4 in PLY with the same binding modality (Fig. 3) except for the distance between Val372 and the fatty chain moiety of the inhibitors, thus highlighting the key residues interacting with the ligands (residues Ala370, Tyr371, Val372, Leu460, and Tyr461). Based on the 3D structures from the MD simulation, it is suggested that the cyclopentaphenanthrene ring of the inhibitors could form a strong hydrophobic interaction with the side chains of Leu460 and Tyr461 in the loop 2 region. In addition, inhibitors were also proximal to residues Ala370, Tyr371, and Val372 (loop 3 region), which suggested a strong interaction between these residues and the fatty chain moieties of the inhibitors. Due to the binding of these inhibitors to the substrate binding region, the cell membrane substrate of PLY, cholesterol, could not bind to PLY, resulting in the loss of lytic activity of PLY. This mechanism has been described by the experiment data in a previous report.

### Confirmation of the PLY binding sites that interact with the inhibitors

**Figure 4:**
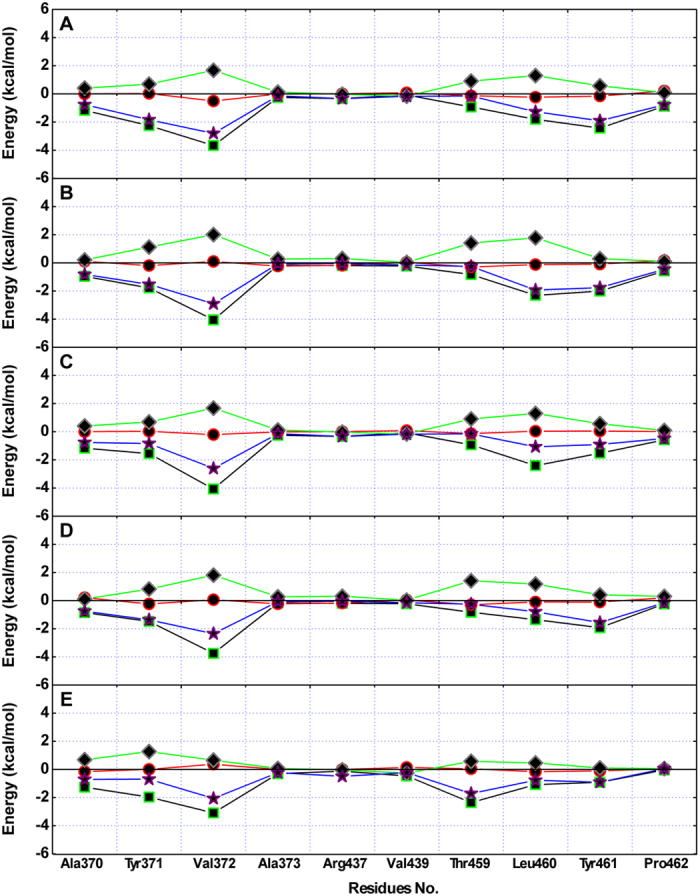
Decomposition of the binding free energy for the residues in the binding sites of PLY. Based on the MM-GBSA method performed for PLY-inhibitor complex systems, Leu460 and Tyr461 have appreciable Van der Waals (*ΔE*_*vdw*_) contributions, and Val372 also has a strong Van der Waals interaction of ≤−2.0 kcal/mol with CHO (**A**), CAM (**B**), SIO (**C**), BRA (**D**) and SIG (**E**).

The binding modes of the five inhibitors of PLY are nearly identical based on the above results; however, the inhibition of haemolytic activity by the inhibitors was quite different based on the results from the haemolysis assay, which indicated that the binding energy of inhibitors with residues in the binding region was different due to the varying molecular structures. To explore the differences among the residues in the binding sites and their contributions to the binding of inhibitors to PLY, inhibitor-residue interaction decompositions based on the MM-GBSA method were performed for the PLY-inhibitor complex systems. Figure 4 shows that Leu460 and Tyr461 have an appreciable Van der Waals (*ΔE*_*vdw*_) contribution due to the close proximity between these residues and the cyclopentaphenanthrene ring of the inhibitors. In addition, Val372 also exerts strong Van der Waals interactions of ≤−2.0 kcal/mol with CHO, CAM, SIO, BRA and SIG. The earlier data indicated that the stabilization of the PLY-inhibitor complexes was mostly due to residues Tyr371, Val372, Leu460, and Tyr461, which has been confirmed by experimental data in a previous report^15^.

### Study of the relationship between the structure and activity of inhibitors

**Figure 5:**
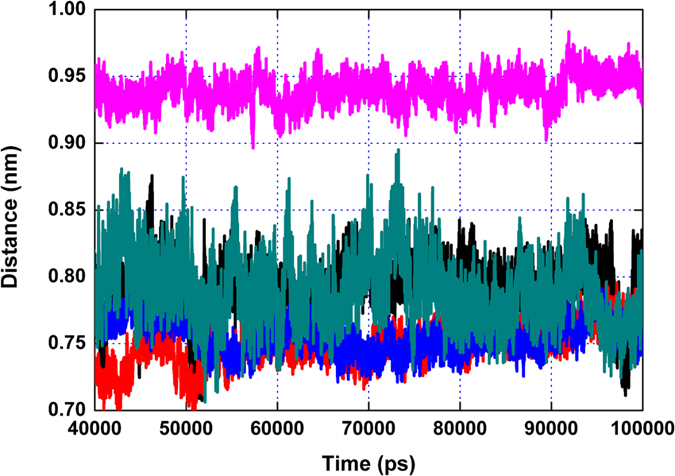
Comparison of the distance between Val372 and the alkyl chain moieties of the ligands in the five complexes. Based on the MD simulation trajectory, the average distances for the PLY-CHO (black line), PLY-CAM (red line), PLY-SIO (blue line), PLY-BRA (dark cyan line), and PLY-SIG (magenta line) complexes were 0.78, 0.70, 0.73, 0.83, and 0.96 nm, respectively.

**Figure 6:**
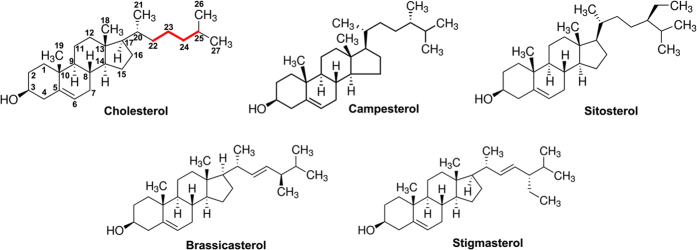
The chemical structures of the five PLY inhibitors. The critical structural distinction of these five natural sterols was the C22-C23-C24-C25 carbon bonds (red lines).

**Figure 7:**
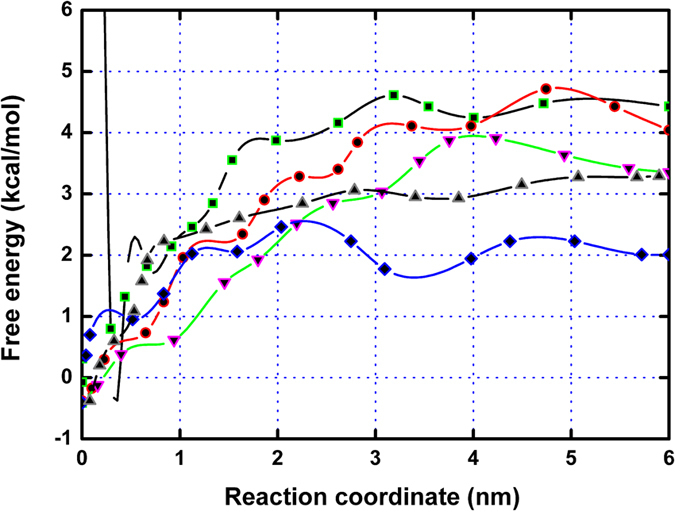
Free energy profiles of the five inhibitors with PLY via the Steered Molecular Dynamics (SMD) method. The calculated binding free energies of the PLY-CHO (square), PLY-CAM (circle), and PLY-SIO (down triangle) complexes were higher than those of the PLY-BRA (diamond) and PLY-LAN (up triangle) complexes.

Val372 is close to the fatty chain moiety of the inhibitors, leading to the existence of an appreciable Van der Waals interaction. However, due to the different distances between Val372 and the fatty chain moieties, the interactions between Val372 and the fatty chain moiety of ERG and LAN were weaker. As shown in Fig. 5, the distance calculation between Val372 and the alkyl chain moiety of the ligands was performed during the MD simulated trajectory. The average distances for the PLY-CHO, PLY-CAM, PLY-SIO, PLY-BRA, and PLY-SIG complexes were 0.78, 0.70, 0.73, 0.83, and 0.96 nm, respectively. Interestingly, the binding free energy between Val372 and the inhibitors decreased in the following order: CHO ≈ CAM ≈ SIO > BRA > SIG, which is consistent with the results of the distance between Val372 and the fatty chain moieties of the inhibitors. It was indicated that the dynamic fluctuations in the distance between Val372 and the alkyl chain moieties of the inhibitors directly affect the values of the binding energy, and the decrease of the binding energy is due to the longer distance between Val372 and the alkyl chain moiety on SIG. Furthermore, the difference in the distance between Val372 and the alkyl chain moieties of the inhibitors was due to whether the alkyl chain moiety was in close proximity to the side chain of Val372 through a single bond (C22-C23-C24-C25) rotation (Fig. 6). As shown in Fig. 3, in the PLY-CHO, PLY-CAM, and PLY-SIO complexes, the alkyl chain moieties were close to the side chain of Val372 and could form a strong Van dal Waals interaction, leading to a stronger binding free energy of PLY with these inhibitors. However, due to the presence of a double bond, the alkyl chain moieties on BRA and SIG could not rotate freely, and the distance between Val372 and the alkyl chain moieties were longer, leading to a weaker binding free energy of PLY with those inhibitors. To confirm this mechanism, the potential of mean force (PMF) was performed to calculate the binding free energies of the five complexes (Fig. 7). The calculated binding free energies of the PLY-CHO, PLY-CAM, and PLY-SIO complexes were ~−4.51 kcal/mol, ~−4.66 kcal/mol, and ~−3.78 kcal/mol, respectively. On the other hand, the calculated binding free energies of the PLY-BRA and PLY-LAN complexes were ~−3.1 kcal/mol and ~−2.53 kcal/mol, respectively. This result is entirely consistent with the proposed mechanism, which indicates that the single bond (C22-C23-C24-C25) alkyl chain moiety is one of the key moieties in the interaction of these inhibitors with PLY.

## Discussion

As is currently known, PLY is a major virulence factor of *S. pneumoniae*^10^,^16^ and can be released as a soluble monomer to kill target cells by assembling into large oligomeric rings and forming pores in cholesterol-containing membranes^8^,^9^,^11^,^17^,^18^,^19^. Currently, PLY has become one of the key candidate targets for the treatment of bacterial infections. In 1983, the role of PLY in the pathogenicity of *S. pneumoniae* was first investigated^20^. When mice were subsequently challenged with virulent *S. pneumoniae* via the nasal route, they survived significantly longer than the control mice. Furthermore, a defined PLY-negative mutant of *S. pneumoniae* was also reported^21^. The study showed that the PLY-negative mutants exerted reduced virulence in mice as judged by the survival time after intranasal challenge, the intraperitoneal 50% lethal dose, and blood clearance studies. In 2014^13^, a vaccine consisting of pneumococcal histidine triad D (PhtD), detoxified pneumolysin derivative (PlyD1), and pneumococcal choline-binding protein A (PcpA) for protection against infection by *S. pneumoniae* was reported. Despite the considerable biomedical interest and effort in studying vaccines and antibodies against PLY, there are few reports on PLY inhibitors.

In our previous study, β-sitosterol, a natural inhibitor of PLY, was found, and the interaction mechanism of PLY with β-sitosterol was explored by the molecular dynamics simulation method^15^. In this work, the discovery of six novel natural compound inhibitors to pneumolysin with differential activity was reported by a haemolysis assay.

Based on the data of the haemolysis assay, it was confirmed that the different molecular structures of the inhibitors lead to differential inhibitory activities. To identify the key chemical moieties of two novel natural pneumolysin inhibitors, the ligand-protein binding sites were surveyed via molecular modelling. Moreover, the MM-PBSA method was used to determine the associated free energy profiles of these interactions. Based on the theoretical calculations performed, the binding modes of five inhibitors of PLY were highly similar, and the stabilization of the PLY-inhibitor complexes was mostly due to residues Tyr371, Val372, Leu460, and Tyr461. However, due to the different distances between Val372 and the fatty chain moieties, the interactions between Val372 and the fatty chain moieties of BRA and SIG were weaker. Based on the analysis of the molecular simulation trajectory, the difference in the distance between Val372 and the alkyl chain moieties of inhibitors was dependent on whether the alkyl chain moiety was in close proximity to the side chain of Val372 through a single bond (C22-C23-C24-C25) rotation. In the PLY-CHO, PLY-CAM, and PLY-SIO complexes, the alkyl chain moieties of these inhibitors were close to the side chain of Val372 and could form the strong Van der Waals interactions, resulting in an increase in binding affinity. However, due to the presence of a double bond, the alkyl chain moieties of BRA and SIG could not rotate freely, and the distance between Val372 and these alkyl chain moieties were longer, leading to a weaker binding free energy of PLY with these inhibitors. These results have been confirmed by potential of mean force (PMF) profiles.

In summary, by the haemolysis assay method, five natural compound inhibitors against pneumolysin with differential activities were discovered, and the interaction mechanism between PLY and these inhibitors was explored using molecular simulations. Based on the theoretical calculation method, the critical chemical feature of a single bond (C22-C23-C24-C25) was found. The inhibitors with this single bond (C22-C23-C24-C25) had higher inhibitory activity compared to BRA and SIG. Thus, it is confirmed that the compounds with a single bond (C22-C23-C24-C25) in the alkyl chain moiety should be good candidates for the design of novel and potent sterol inhibitors of PLY.

## Materials and Methods

### Bacteria, chemicals and pneumolysin purification

*S. pneumoniae* strain D39, which was kindly provided by Dr. David E. Briles (Departments of Microbiology, University of Alabama at Birmingham), was cultured at 37 °C using Todd-Hewitt broth. The chemicals (cholesterol, campesterol, sitosterol, brassicasterol and stigmasterol) used in our study were commercially purchased from Sigma-Aldrich (St. Louis, MO, USA). The minimal inhibitory concentrations (MICs) of the five natrual compounds for D39 were identified using the broth microdilution method based on the Clinical and Laboratory Standards Institute (CLSI) guidelines. The cloning, expression and purification of pneumolysin was performed as previously described^15^.

### Haemolysis assay

Approximately 10 μl of purified pneumolysin (100 μg/ml) was incubated in microtiter plates at 37 °C for 15 min with serial dilutions of the tested sterols. A volume of 50 μl (5 × 10^6^ cell/ml) of defibrinated sheep blood in PBS was added to the wells, and the final volume of the reaction was adjusted to 1 ml with PBS. The reactions were incubated at 37 °C for 25 min. Reactions that received 10 μl of 1% Triton X-100 and PBS were used as positive and negative controls, respectively.

### Initial model construction

Because the monomeric crystal structure of pneumolysin (PLY) is unavailable, the 3D structure of PLY was constructed based on the template structures of perfringolysin O (PDB code: 1 M3I) using a homology simulation method. The detailed calculations were based on previous reports in the literature^22^,^23^,^24^. Thus, standard molecular docking was performed using the 3D structure of PLY gained from the homology simulation study as the receptor and the five inhibitors as the flexible ligands based on the Autodock4.0 package.

### Molecular simulation

A standard MD simulation was performed using Gromacs 4.5.4 biomolecular simulation software^25^ and Amber99sb force field. The TIP3P water box^26^ with a distance between the protein molecule and the periodic box boundary of at least 0.8 nm was used to solve the PLY-inhibitor complex systems. Initial coordinates of the protein-inhibitor complexes were extracted from the molecular docking data. For each simulation, the energy minimization was first performed allowing for the movement of water molecules and neutralizing ions until convergence to 100 kJ/(mol·nm). The detailed process of the MD simulation was reported in our previous publications^22^,^23^. For the inhibitors (used as ligands), the parameters were estimated by the antechamber programs and AM1-REST partial atomic charges in the Amber10 software package.

### Calculation of the Binding Free Energy

The MM-PBSA^27^,^28^,^29^,^30^ approach supplied by the Amber 10 package was used to calculate the binding free energy between the protein and ligand. The detailed calculation was based on previous reports in the literature^22^,^24^.

### Steered Molecular Dynamics (SMD)

During the SMD simulation process, to gain insight into the processes of inhibitor binding with PLY, the external force was directly applied to the ligands, and potential of mean force (PMF) was calculated via umbrella sampling method based on the Gromacs 4.5.5 software. The detailed calculation was based on previous reports in the literature^24^.

## Additional Information

**How to cite this article**: Li, H. *et al*. Insights into structure and activity of natural compound inhibitors of pneumolysin. *Sci. Rep.* **7**, 42015; doi: 10.1038/srep42015 (2017).

**Publisher's note:** Springer Nature remains neutral with regard to jurisdictional claims in published maps and institutional affiliations.
